# Distinct EEG Features and Sleep/Wake Behaviour Predict Anxiety Phenotypes in Mice

**DOI:** 10.1111/jsr.70425

**Published:** 2026-07-30

**Authors:** A. Altunkaya, K. M. Mengel, M. V. Schmidt, B. Solak, M. Kreuzer, G. Rammes, G. Schneider, T. Fenzl

**Affiliations:** ^1^ Clinic for Anesthesiology and Intensive Care, School of Medicine and Health Technical University of Munich Munich Germany; ^2^ Research Group Neurobiology of Stress Resilience Max Planck Institute for Psychiatry Munich Germany

**Keywords:** brain waves, electrophysiology, polysomnography, rodent, sleep–wake behaviour, vigilance states

## Abstract

Preoperative anxiety is a prevalent clinical problem linked to adverse surgical outcomes, including elevated pain, prolonged hospital stays and increased risk of postoperative delirium. Current assessments rely heavily on subjective measures, necessitating objective biomarkers. We investigated whether baseline electroencephalography (EEG) and sleep–wake profiles can predict anxiety levels in a mouse model. Fifty male C57BL/6N mice were implanted with EEG and electromyography (EMG) electrodes, recorded for 24 h post‐recovery before undergoing a cued fear‐conditioning protocol. Six behavioural indices, capturing freezing during tone and intertone epochs in both conditioning and retrieval, were used in k‐medoids clustering to classify mice into high‐anxiety or low‐anxiety groups. Comparative analysis revealed pronounced group differences in baseline sleep architecture. HA mice exhibited diminished rapid eye movement sleep (REMS) proportions and elevated NREMS in the dark period, alongside altered bout lengths. Spectral analysis underscored lower delta (0.5–2 Hz) power in high‐anxiety mice, with heightened eta/beta (~15–30 Hz) activity during REMS suggesting aberrant cortical arousal. Moreover, high‐anxiety mice showed significantly longer and higher‐amplitude sleep spindles, reinforcing the interplay between disrupted sleep microstructure and enhanced anxiety‐like responses. These findings illustrate that specific EEG and sleep–wake parameters prior to a conditioned stimulus can forecast distinct anxiety phenotypes in a widely used inbred mouse model. Although our study focuses on fundamental mechanisms in laboratory animals, this line of research may lead to objective markers of anxious behaviour susceptibility in broader contexts, potentially identifying individuals at higher risk for heightened anxiety in clinically relevant settings, for example, preoperative anxiety.

## 1 Introduction

Anxiety disorders are among the most prevalent psychiatric conditions, imposing a substantial burden on affected individuals and healthcare systems alike (WHO 2025). A growing body of evidence suggests that susceptibility to anxiety is not solely determined by acute environmental stressors but is in part shaped by trait‐like predispositions that vary across individuals (Clauss and Blackford 2012). These predispositions, rooted in stable neurobiological differences, may manifest in baseline neurophysiological activity, particularly, in sleep architecture and electroencephalographic (EEG) patterns, even before exposure to anxiogenic stimuli (Cox and Olatunji 2020). The hypothesis that such baseline signatures could serve as predictive indicators of anxiety vulnerability has gained traction, yet direct empirical evidence linking pre‐existing sleep and EEG features to subsequent anxiety‐like responses remains limited (Bush et al. 2022; Cox and Olatunji 2020).

One promising avenue for testing this hypothesis lies in the investigation of sleep disturbances and their neurophysiological underpinnings. Such disturbances are frequently observed in individuals with anxiety disorders (Wang et al. 2020). Alterations in sleep architecture such as reduced total sleep time, decreased slow‐wave sleep and diminished rapid eye movement sleep (REMS) proportions have been consistently associated with elevated anxiety levels (Cox and Olatunji 2020). Electroencephalography (EEG) offers a non‐invasive method to assess neural activity during sleep and wakefulness, providing potential insights into the neurophysiological correlates of anxiety (Schutter and Knyazev 2012). Additionally, the EEG may also serve as a predictive tool, showing latent electrophysiological patterns that can forecast subsequent anxiety symptom severity and diagnostic outcomes (Bosl et al. 2023).

One clinical domain where objective anxiety assessment is, particularly, needed is preoperative anxiety (POA). POA represents a prevalent issue among patients awaiting surgical procedures, characterised by heightened feelings of stress, fear and apprehension about the operation and its outcomes (Verhoeven et al. 2024). A significant proportion of patients are affected, with reported prevalence rates ranging from 11% to 80% depending on the type of assessment method and patient population (Aust et al. 2018; Corman et al. 1958; Norris and Baird 1967). Current methods for assessing POA predominantly rely on subjective self‐report tools (Julian 2011), which can be susceptible to bias, as patients may underreport their symptoms (Eberhart et al. 2020). Objective assessment tools that can reliably identify POA are urgently needed (Kim et al. 2023).

To investigate the neurobiological underpinnings of anxiety and its association with sleep–wake behaviour, animal models provide an invaluable platform. Rodents, in particular, allow for controlled experimental manipulation of variables that are not feasible in human studies (Fenzl et al. 2011; Gencturk and Unal 2024). Mouse models are advantageous due to their exhibition of measurable anxiety‐like behaviours (Jakubcakova et al. 2012) and the possibility for in vivo electrophysiological investigations under controlled conditions, not possible in human patients (Buzsaki 2019). Moreover, EEG recordings in mice can provide detailed information about brain activity during different vigilance states, including wakefulness, non‐REM sleep (NREMS) and REMS (Vyazovskiy et al. 2009).

In rodents, fear conditioning (FC) paradigms have been employed for more than five decades to measure fear responses (Blanchard and Blanchard 1969) and can be utilised to assess anxiety‐like behaviours (Walker et al. 2009). In these paradigms, mice learn to associate a neutral stimulus, such as a tone, with an aversive event like a mild foot shock, leading to the expression of conditioned fear responses upon subsequent presentations of the tone (McGuire et al. 2012). This behavioural paradigm closely parallels aspects of human anxiety and is sensitive to both innate and situational anxiety traits (Gordon and Adhikari 2010). Despite advances in understanding the behavioural and physiological aspects of anxiety, the relationship between baseline neurophysiological patterns, particularly, those observed in EEG recordings and sleep–wake behaviour and anxiety levels remains incompletely understood.

Addressing this gap, the present study explores the potential of baseline EEG activity and sleep–wake patterns as prognostic measures that may predict the level of anxiety‐like behaviour in mice. Establishing reliable baseline indicators could not only advance fundamental understanding of anxiety predisposition but also contribute to the development of objective assessment tools in clinical contexts such as POA.

## 2 Materials and Methods

### 2.1 Animal Model and Housing

For this study, 50 male C57BL/6N mice (Charles River Laboratories, Germany), aged 12–16 weeks, were used. The care of laboratory animals and all experiments were performed in accordance with the recommendations of the European Union and the ARRIVE guidelines 2.0 (Percie du Sert et al. 2020). All experimental protocols received authorisation from the Bavarian Government (ROB‐55.2‐2532.Vet_02‐21‐73). The study was not preregistered with the Open Science Framework. Mice were housed individually in recording cages within sound‐attenuated chambers on a 12 h light–dark cycle (lights on at 06:00, ZT0) at 22°C ± 2°C and 55% ± 10% humidity. Food and water were provided ad libitum. The specific details of the housing, enrichment and husbandry protocols were identical to those described in previous work from our laboratory (Altunkaya et al. 2024; Joyce et al. 2024).

### 2.2 EEG/EMG Electrode Assembly

Electrode‐sockets incorporating EEG and electromyographic electrodes (EMG) were assembled using a gold wire (751 GG gold wire, ø 150 μm; C. Hafner, Wimsheim, Germany) soldered onto 8‐pin PCB‐sockets (PRECI‐DIP SA Series 861, Delémont, Switzerland)(Altunkaya et al. 2024). Each socket accommodated two EEG electrodes, two EMG electrodes and one ground electrode, leaving the remaining three pins unused. To enhance structural rigidity, a dental cement cover (Paladur, Heraeus‐Kulzer, Hanau, Germany) was applied to the soldering side of the PCB‐socket.

### 2.3 Surgery for Electrode Implantation

For electrode implantation, mice were anaesthetised with isoflurane (3% induction, 1.4%–2.0% maintenance) in a stereotactic frame. Body temperature was maintained at 37°C using a feedback‐controlled heating pad. Following subcutaneous application of lidocaine, two epidural EEG electrodes were implanted over the right frontal and right occipital cortices (see Figure 1a for coordinates) and two EMG electrodes were inserted into the bilateral *musculus semispinalis capitis*. The head‐mount assembly was affixed to the cranium using jeweller's screws and dental cement. Postoperative care included analgesia with carprofen and monitoring until full recovery. The detailed surgical procedures, including all materials and anaesthetic protocols, have been described previously (Altunkaya et al. 2024).

**FIGURE 1:**
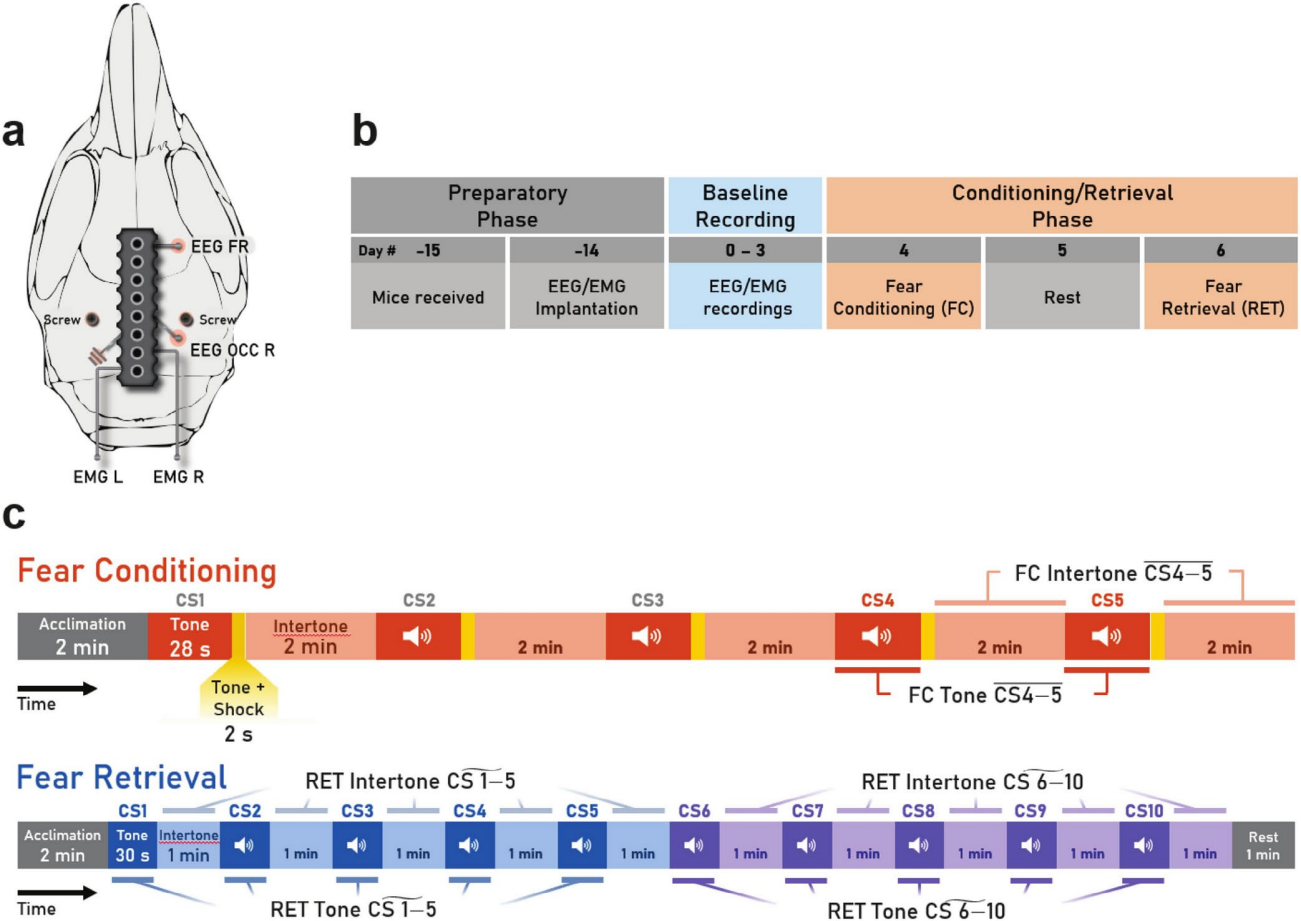
Experimental timeline and behavioural readout design. (a) Implanted PCB socket along with implantation locations. Electrodes were labelled as follows: EEG FR (right frontal EEG), EEG OCC R (right occipital EEG), ground electrode (depicted with ground symbol) and the left and right EMG electrodes, as well as two screws used for skull mounting. The coordinates are as follows: EEG FR: mediolateral (ML): 1 mm, anterior–posterior (AP): 2.8 mm, dorsoventral (DV): −0.5 mm EEG OCC R: ML: 2.8 mm, AP: −3.5 mm, DV: −0.5 mm Ground: ML: −2.8 mm, AP: −3.5 mm, DV: −0.5 mm. The two EMG electrodes were implanted bilaterally. (Note: Illustration proportions are not to scale; mouse skull adapted from Cook (1965)). (b) Experimental timeline for EEG and fear conditioning. Mice undergo surgery (Day # −15) and have a 2‐week recovery. Basal EEG recording (Day 0, blue) marks the start of experiment, followed by fear conditioning (Day 4, green) and a rest day (Day 5). Fear retrieval (Day 6, green) is tested. (c) Schematic of the fear conditioning and retrieval protocols. During fear conditioning, mice experienced five tone (CS) presentations (CS1‐5), each co‐terminating with a mild foot shock, separated by intertone intervals. On retrieval day, mice were re‐exposed to 10 tone presentations (CS1‐10) without shock, separated by 1‐min intertone intervals. The durations were defined as follows: FC tone: 28 s, FC intertone interval: 120 s, RET tone: 30 s and RET intertone interval: 60 s. Analyses focused on six key behavioural measures: The average freezing during the last two‐tone presentations ($\mathrm{FC}\;\text{Tone}\;\bar{\mathrm{CS}4-5}$) and their intertone intervals ($\mathrm{FC}\;\text{Intertone}\;\bar{\mathrm{CS}4-5}$). The first five ($\mathrm{RET}\;\text{Tone}\;\tilde{\mathrm{CS}\;1-5}$, $\mathrm{RET}\;\text{Intertone}\;\tilde{\mathrm{CS}\;1-5}$) and last five ($\mathrm{RET}\;\text{Tone}\;\tilde{\mathrm{CS}\;6-10}$, $\mathrm{RET}\;\text{Intertone}\;\tilde{\mathrm{CS}\;6-10}$) were analysed by taking the median freezing proportion.

### 2.4 EEG/EMG Recordings

After a 14‐day recovery period, the PCB socket was connected to a recording system consisting of a commutator and a rotatable, weight‐balanced swivel system, as previously described (Altunkaya et al. 2024). The raw EEG signal was amplified (INA128 instrumentation amp, combined with an OPA244 non‐inverting stage, gain ≈10,000×) and analog band‐pass filtered (0.5–120 Hz, fifth‐order high‐pass/third‐order low‐pass) by a custom front end. The EMG channel used the same architecture with filter corners at 10 Hz and 12 kHz but was amplified by a factor of 1000. Both signals were digitised with 16‐bit resolution and a sampling rate of 256 Hz (NI USB‐6343). All signals were recorded continuously using EGErA recording software. EEG recordings were initiated at ZT0 and continued for 72 h (Figure 1b) and the last 24‐h period was selected for subsequent data analysis. Mice were recorded in multiple batches, with all basal recordings conducted simultaneously within batches.

### 2.5 Cued FC and Fear Retrieval

Cued FC and fear retrieval (RET) tests were employed to assess and classify anxiety phenotypes in mice (Figure 1c). Experiments were conducted in an isolated room, away from the housing area, using a FC apparatus (FREEZING AND STARTLE Threshold Sensor including Sound Attenuating Box, LE116 76‐0280, Panlab, Barcelona, Spain). The apparatus comprised a cage measuring 15 × 15 × 25 cm (width × depth × height) with the entrance door including a cutout covered with a red‐filter acrylic glass. The floor consisted of a metal grid for electric foot‐shock administration and the cage top featured a circular opening for video camera mounting confined within the sound attenuated box.

The FC setup was controlled using Packwin software (Panlab, Barcelona, Spain). The FC protocol initiated with a 2‐min acclimatisation period for basal behaviour assessment, followed by a 30‐s epoch of auditory stimulus (conditioned stimulus, CS; 10 kHz, 75 dB). During the final 2 s of the CS, a co‐terminating foot shock (unconditioned stimulus, US; 0.6 mA) was administered. This was succeeded by a 2‐min intertone interval without auditory or shock stimuli. This sequence was iterated five times, yielding five tone phases and four intertone intervals and concluded with a 2‐min consolidation phase, which is counted as the fifth and last intertone interval.

The RET test was performed 24 h post‐FC, at the same time and location (ZT14). The RET protocol commenced with a 2‐min acclimatisation phase, followed by a 30‐s epoch of the CS (10 kHz, 75 dB), without the US. A 1‐min intertone interval without auditory stimulus followed. The RET sequence was repeated 10 times, resulting in 10 tone intervals and 10 intertone intervals, terminating with a 1‐min consolidation phase.

Throughout both FC and RET procedures, behaviour was recorded using a camera at 1920 × 1080 resolution with a frame rate of 30 fps (GoPro Inc., San Mateo, CA, USA). The resulting video data underwent analysis for freezing behaviour by using a custom MATLAB script (R2024b, MathWorks, Natick, MA, USA).

### 2.6 Data Analysis: Anxiety Phenotyping

Analyses focused on six key behavioural measures:

1. $\mathrm{FC}\;\text{Tone}\;\bar{\mathrm{CS}4-5}$: average freezing % during FC tone CS4‐5
2. $\mathrm{FC}\;\text{Intertone}\;\bar{\mathrm{CS}4-5}$: average freezing % during FC intertone CS4‐5
3. $\mathrm{RET}\;\text{Tone}\;\tilde{\mathrm{CS}\;1-5}$: median freezing % during RET tone CS1‐5
4. $\mathrm{RET}\;\text{Intert}\mathrm{one}\;\tilde{\mathrm{CS}\;1-5}$: median freezing % during RET intertone CS1‐5
5. $\mathrm{RET}\;\text{Tone}\;\tilde{\mathrm{CS}\;6-10}$: median freezing % during RET tone CS6‐10
6. $\mathrm{RET}\;\text{Intert}\mathrm{one}\;\tilde{\mathrm{CS}\;6-10}$: median freezing % during RET intertone CS6‐10

Normal distribution was assessed using the Shapiro–Wilk test. To see whether any of these six measures naturally form multiple clusters, multimodality of data distribution was analysed. For this, Hartigan's dip test was applied, quantifying the deviation of each behavioural measure's empirical distribution from unimodality. The statistical significance of the dip statistic was evaluated via bootstrap procedure with 10,000 iterations.

K‐medoids clustering, a robust partitioning algorithm that groups data based on pairwise dissimilarities using representative data points (medoids), with *k* = 2 was chosen to classify mice into low anxiety (LA) and high anxiety (HA) groups (see also Figure S2). The Manhattan distance metric was selected to minimise potential skewing during clustering (Chugani 2024) and 100 replicates each with a maximum of 10,000 iterations were used to ensure convergence. To assign LA versus HA labels, the median freezing proportion was determined for each cluster based on an unscaled copy of the cleaned data.

To assess how well‐separated the LA and HA groups were, we computed silhouette scores, which measure how similar each mouse is to its own cluster relative to the nearest other cluster (silhouette function in MATLAB; R2024b, MathWorks, Natick, MA, USA). The silhouette value $s_{i}$ for the ith point is defined as

$$s_{i}=\left(b_{i}-a_{i}\right)/\max\left(a_{i},b_{i}\right)$$

where $a_{i}$ is the average distance from the ith point to the other points in the same cluster as i and $b_{i}$ is the minimum average distance from the ith point to points in a different cluster, minimised over clusters (Kaufman et al. 1990).

After confirming the cluster stability, non‐metric multidimensional scaling (NMDS) was carried out to project the observations into two dimensions for data visualisation purposes. A cityblock distance metric was chosen for the pairwise distance matrix calculation. The optimisation used a stress‐based criterion to comply with our non‐parametric dataset in two‐dimensional (2D) space and was executed with 100 replicates of random starting configurations, each constrained to a maximum of 10,000 iterations. Following NMDS, the stress value was calculated to quantify how well the distances in the 2D representation approximate the original pairwise distances in the six‐dimensional data.

In order to approximate factor loadings analogous to those typically obtained from principal component analysis, each original variable was correlated using Pearson's correlation with both NMDS dimensions. This correlation‐based measure was treated as a vector, anchoring at the origin and extending in the direction determined by the sign and magnitude of the correlation with each axis. These vectors were then overlaid onto the 2D NMDS plot to highlight how strongly each variable aligned with the derived dimensions.

### 2.7 Data Analysis: EEG

All EEG/EMG recordings were analysed using custom‐made MATLAB scripts and LabView (LabVIEW 2014, National Instruments, Austin, Texas) (Kreuzer et al. 2015). Raw data were downsampled to 125 Hz and divided into light (ZT0‐12) and dark (ZT12‐24) periods. Vigilance states were scored in 4‐s epochs by two experts, blinded to the subsequent anxiety group assignments, using semi‐automated software (Kreuzer et al. 2015), with each epoch being manually reviewed and corrected as needed. From the scored data, temporal features (vigilance state proportions, bout lengths, transition probabilities, latencies) and spectral features (power spectral density [PSD]) were calculated. The detailed methodology for sleep scoring, artefact handling and the calculation of all temporal and spectral parameters was identical to our previous work (Altunkaya et al. 2024; Fritz et al. 2021).

### 2.8 Data Analysis: Sleep Spindle Detection

Analysis was performed using an automated MATLAB‐based paradigm for the detection of sleep spindles (SPs) from mouse EEG recordings (Uygun et al. 2018). Detection parameters: minimum SP duration: 0.5 s, maximum SP duration: 2 s, inter‐SP interval: 0.1 s. Raw EEG traces were first bandpass filtered between 10 and 15 Hz (Butterworth band‐pass filter: first stopband frequency: 3 Hz, first passband frequency: 10 Hz, second passband frequency: 15 Hz, second stopband frequency: 22 Hz, stopband attenuation 24 dB/octave). The root mean square (RMS) of the filtered EEG was calculated using a 750 ms window. RMS values were then cubed to enhance the signal‐to‐noise ratio on the y‐axis and facilitate threshold definition. A two‐threshold approach was used to establish inclusion criteria for SP detection during NREMS (lower threshold: 1.0 mean cubed RMS; upper threshold: 2.5 mean cubed RMS). SP were analysed for duration, normalised amplitude and SP density (spindle amount divided by NREMS duration [Spindle/min]). Normalised amplitude was calculated by dividing the peak value of the rectified signal envelope by the upper SP detection threshold.

### 2.9 Statistical Analyses

Statistical analyses of all temporal, spectral, SP results were performed using MATLAB. Unless otherwise stated, statistical analyses were performed using the Mann–Whitney test for cross‐sectional comparisons or the Wilcoxon signed‐rank test for longitudinal comparisons.

For the evaluation of differences in spectral power features, calculation of the area under the curve (AUC) of the receiver‐operating characteristic (ROC) for each bin with a frequency resolution [(125/512) Hz] of PSD and 10 k‐fold bootstrapped 95% CIs were performed using the MATLAB‐based MES toolbox (Hentschke and Stuttgen 2011). For vigilance state over time, the AUC and 95% CI values for each 2‐h bin were computed. AUC was defined as a strong classifier if the AUC > 0.7 or AUC < 0.3 and if the 95% CIs did not include 0.5, which also led to the difference between two distributions being considered as significant (Hentschke and Stuttgen 2011). AUC was defined as a good classifier if the AUC > 0.7 or AUC < 0.3 irrespective of 95% CI range (Mandrekar 2010). The significance level was set to *p* < 0.05. All data are presented as individual data points and/or median and CIs unless stated otherwise. All significant and non‐significant statistical data along with AUC effect size calculations are listed in Table S1.

## 3 Results

Analysis of six behavioural readout phases showed differing distribution patterns (Figure 2a, for a table of descriptive statistics, normality test and multimodality test, see Figure S1 and Table S2). A Kruskal–Wallis test revealed a significant overall effect of condition on freezing (*χ* ^2^(5) = 5.00, *p* < 0.0001). Dunn–Sidak‐corrected post hoc comparisons indicated that freezing during FC Tone CS4‐5 was significantly greater than FC Intertone CS4‐5 (*p* = 0.0343), RET Intertone CS1‐5 (*p* = 0.0003) and RET Tone CS6‐10 (*p* = 0.0005). No other pairwise differences reached significance (for a table of all comparison results, see Table S1).

**FIGURE 2:**
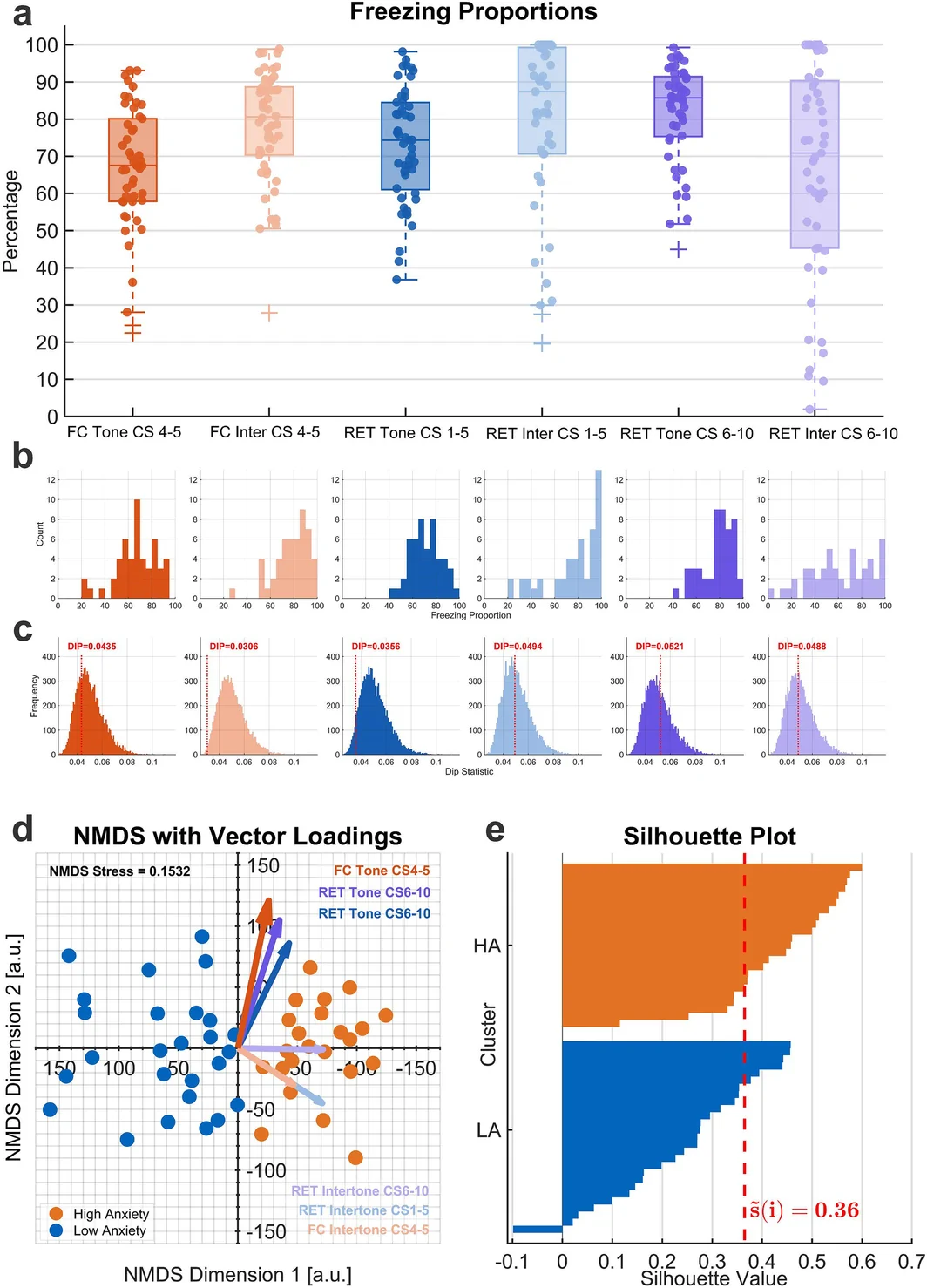
Summary of freezing proportions, clustering analysis and stability testing in fear conditioning and retrieval tasks. (a) Box plot of freezing proportions across six behavioural readout phases. Each box represents the distribution of freezing percentages (%), with individual data points overlaid. Boxes display the interquartile range (IQR) with median values marked and whiskers indicate 1.5× IQR. (b) Histograms of freezing proportions for the six behavioural readout phases. (c) Hartigan's dip test results. Histograms show the bootstrapped distribution of the dip statistic under the null hypothesis of unimodality, along with the empirical dip obtained from the dataset. (d) Behavioural phenotype classification results using k‐medoids clustering. For visualisation purposes, non‐metric multidimensional scaling (NMDS) was used to represent the data in 2D space. A stress value of 0.1532 indicated an acceptable but moderately distorted representation. Spearman‐correlation‐based vector loadings for each readout phase were overlaid to highlight their contributions to NMDS. Points are colour‐coded by cluster (blue: LA, orange: HA). (e) Silhouette plot for analysing the stability of k‐medoids clustering. Bars represent silhouette values for individual data points, stratified by HA and LA clusters. The red dashed line marks the median silhouette value ($\tilde{s}\left(i\right)=0.36$), indicating a moderate clustering quality.

Shapiro–Wilk test results showed that all phases other than RET Tone CS1‐5 and RET Intertone CS6‐10 significantly deviated from normality. Visual inspection of the individual distributions did not suggest bimodal or multimodal patterns (Figure 2b) and Hartigan's dip test confirmed that all six phases were distributed unimodally (all *p* > 0.05; Figure 2c, Table S2). To build two behavioural phenotype clusters—namely LA and HA—with the least amount of median absolute deviation, a k‐medoids clustering approach was employed, which resulted in 27 mice being assigned as the LA group and 23 as HA (Figure 2d).

Spearman‐correlation‐based vector loadings originating from tone phases showed orthogonality to the vector loadings from intertone phases, indicating a distinctive effect on the clustering irrespective of which day the behaviour was recorded from, that is, FC or RET (Figure 2d). FC and RET intertone phases explained the variance of first NMDS dimension to a greater extent, whereas FC/RET tone phases had a more equal contribution along both NDMS dimensions.

The stability of the two‐cluster solution was evaluated via silhouette analysis (Figure 2e), with median silhouette values of 0.2713 for LA and 0.4582 for HA (total median silhouette value = 0.3646), indicating a moderate stability of the clusters (Kaufman et al. 1990). No additional significant effects emerged for these FC and RET outcomes beyond those noted above.

After classifying the mice into LA and HA groups, a retrospective comparison of basal sleep/wake behaviour features, recorded before FC was performed. Representative 10‐s EEG traces from one LA and one HA mouse, illustrating the raw signal morphology during wakefulness, NREMS, REMS and a detected SP, are shown in Figure 3. During basal conditions, LA and HA mice showed a multitude of sleep–wake behaviour differences (Figure 4a–c). Notably, REMS proportion in the light phase was significantly lower for HA mice (*p* = 0.004, AUC = 0.742, 95% CI: 0.596–0.868, Figure 4c,d). In the dark phase, WAKE % (*p* = 0.024, AUC = 0.688, 95% CI: 0.536–0.823, Figure 4e) showed a significant drop in HA mice, whereas NREMS (*p* = 0.041, AUC = 0.330, 95% CI: 0.188–0.485, Figure 4e) proportions were increased. Bout lengths during dark phase were significantly shorter for HA mice (*p* = 0.034, AUC = 0.678, 95% CI: 0.520–0.827, Figure 4i). Transition percentages indicated that HA mice had a significantly higher NREMS‐to‐WAKE transition proportion in the light phase (*p* = 0.032, AUC = 0.322, 95% CI: 0.174–0.486, Figure 4l). Latency measures were largely unchanged; however, HA mice had a significantly shorter WAKE latency in the dark‐phase (*p* = 0.014, AUC = 0.704, 95% CI: 0.552–0.841, Figure 4g).

**FIGURE 3:**
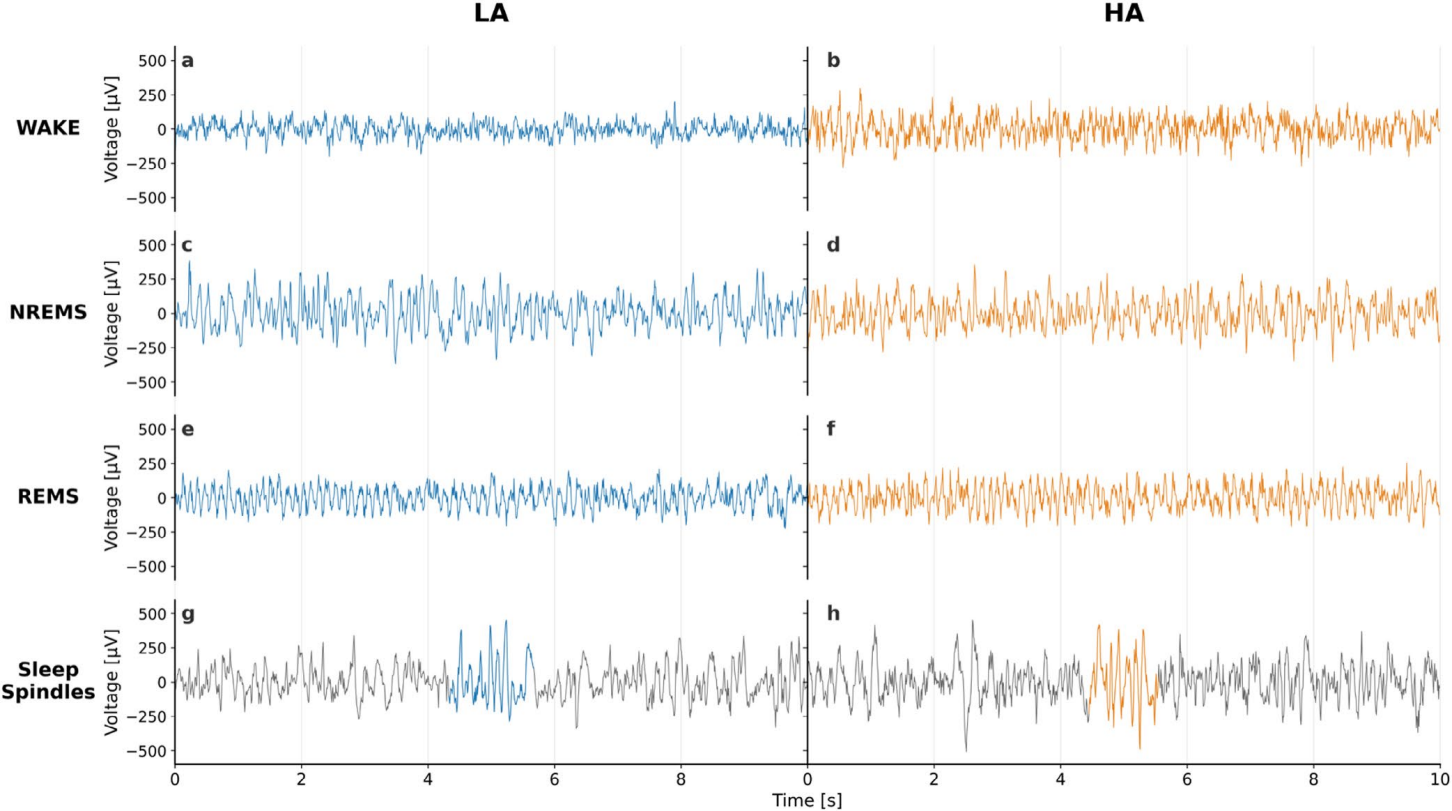
Representative 10‐s EEG traces from one LA mouse (blue) and one HA mouse (orange) during baseline recording. (a, b) Wakefulness. (c, d) NREMS. (e, f) REMS. (g, h) SP during NREMS, with the detected SP highlighted in the respective group colour. Left column (a, c, e, g): LA mouse; right column (b, d, f, h): HA mouse. All panels share the same voltage scale.

**FIGURE 4:**
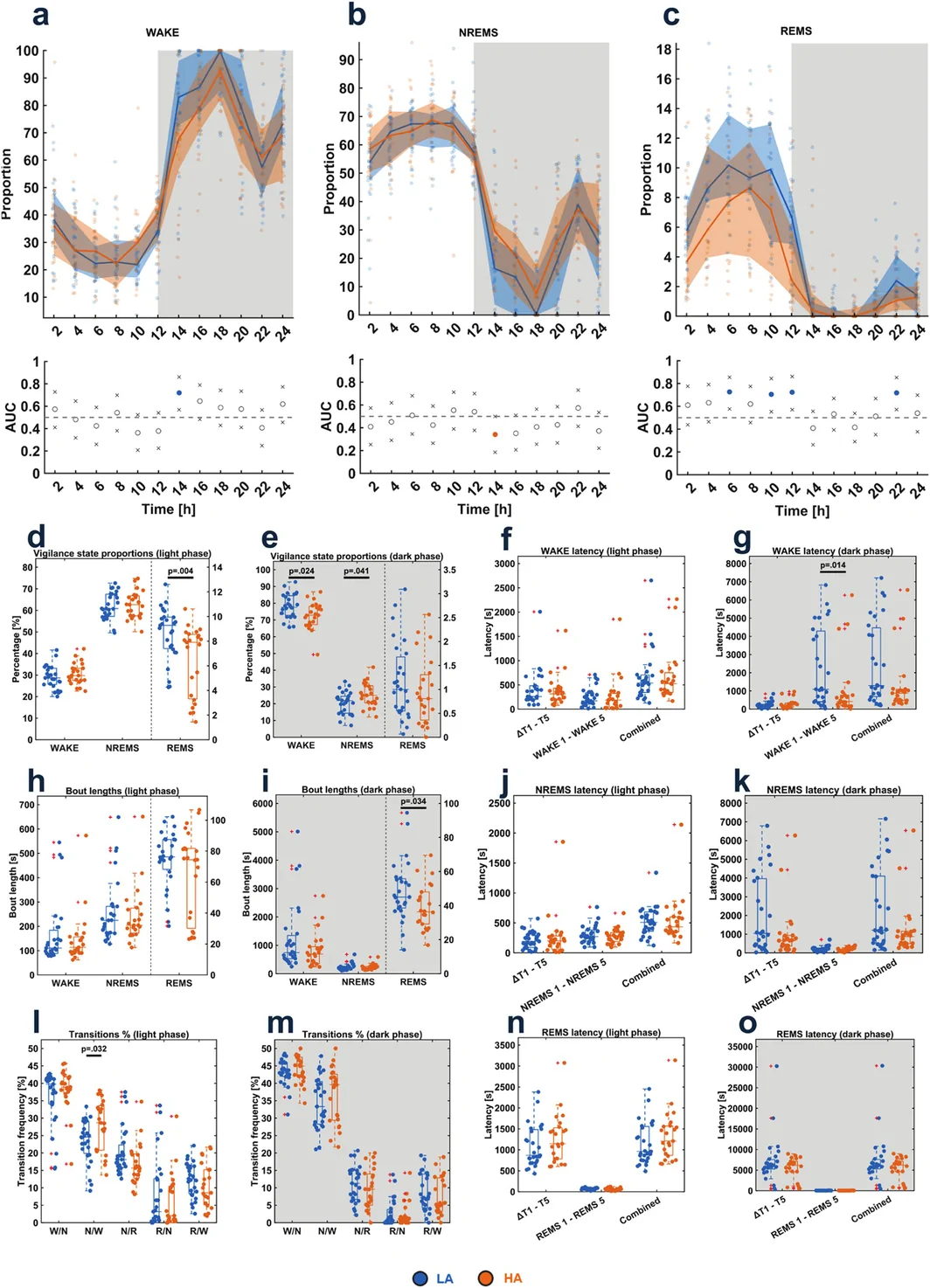
Sleep–wake analysis comparing LA (blue) versus HA (orange) across different vigilance states and parameters. Time course of wake (a), NREMS (b) and REMS (c) proportions across the 24‐h cycle, with shaded areas indicating the dark phase. Data points represent individual values, lines show mean trends and error bars indicate variability. Below each plot, area under the curve (AUC) values are displayed across time bins. Vigilance state proportions during the light (d) and dark (e) phases. Wake latency in the light (f) and dark (g) phases. Bout lengths for wake, NREMS and REMS during the light (h) and dark (i) phases. NREMS latency in the light (j) and dark (k) phases. Percentage of vigilance state transitions in the light (l) and dark (m) phases, including transitions between WAKE (W), NREMS (N) and REMS (R). REMS latency during the light (n) and dark (o) phases. Data are represented as scatter plots with box‐and‐whisker overlays indicating group distributions.

Spectral features, depicted by PSD curves for each vigilance state, showed distinct differences in relative power both within light and dark phase (Figure 5a–f). During light phase, HA mice had a significantly higher power between 14 and 16 Hz during WAKE (Figure 5a; AUC = 0.32 [0.17–0.49]), whereas NREMS power between 0.5–2 Hz range was significantly lower in HA mice compared to their LA littermates (Figure 5b; AUC = 0.70 [0.57–0.83]). In particular, during light phase REMS, 20–30 Hz power displayed a significant increase in HA mice (Figure 5c; AUC = 0.31 [0.16–0.46]). Dark phase vigilance state PSDs showed similar patterns, with a significant decrease in 0.5–2 Hz power during NREMS (Figure 5e; AUC = 0.70 [0.56–0.84]) and an increase in 16–28 Hz during REMS in HA mice (Figure 5f; AUC = 0.32 [0.15–0.49]). WAKE PSDs showed no significant difference between LA and HA during dark phase (Figure 5d). The remaining frequency intervals showed no significant change throughout the 24‐h basal EEG recording.

**FIGURE 5:**
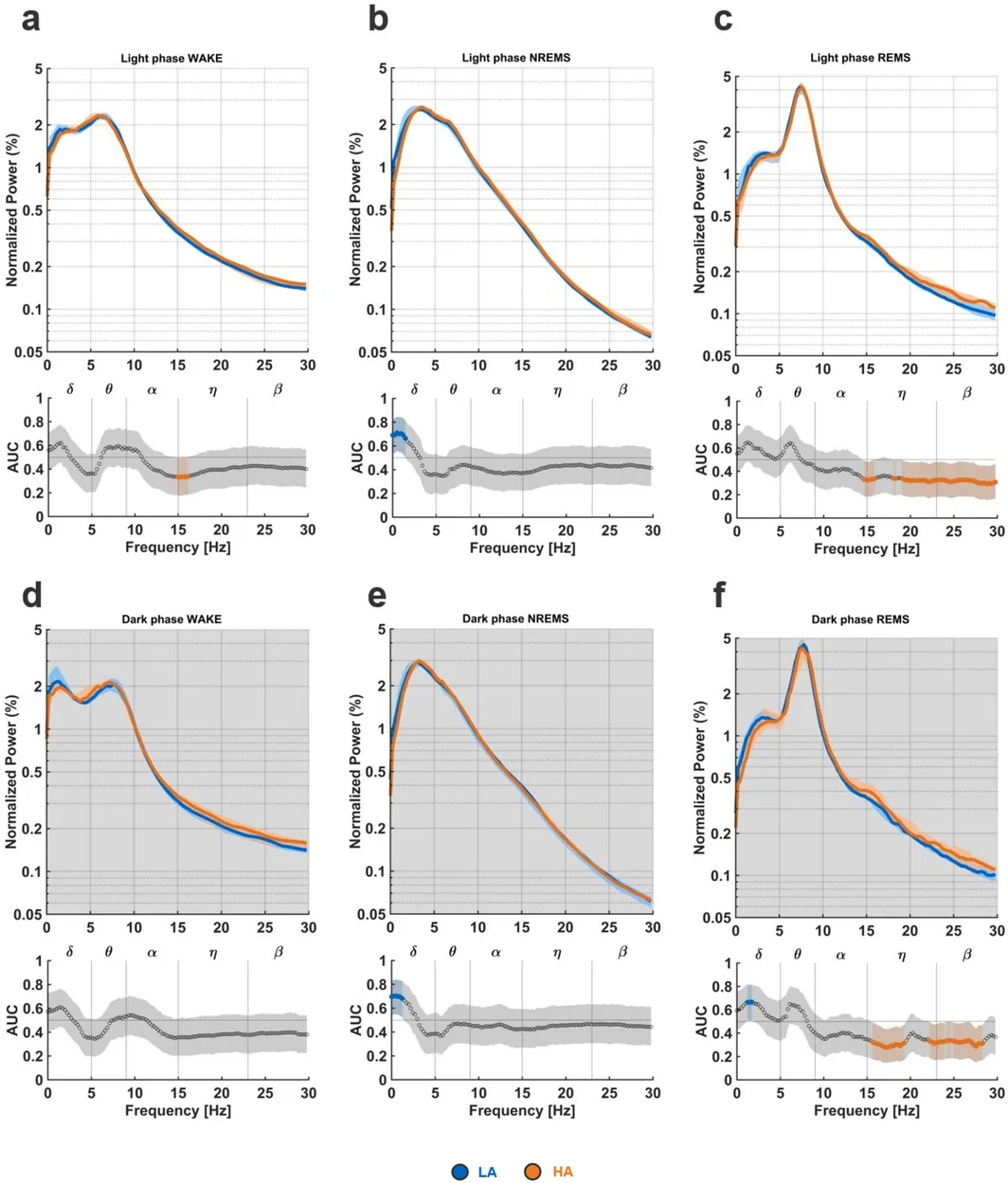
Power spectral density (PSD) analysis of EEG activity during WAKE, NREMS and REMS across light and dark phases for LA (blue) and HA (orange). Normalised power spectra during the light phase for wake (a), NREMS (b) and REMS (c), with corresponding area under the curve (AUC) plots below. Normalised power spectra during the dark phase for wake (d), NREMS (e) and REMS (f), with AUC plots below. The *x*‐axis represents frequency (0–30 Hz), while the *y*‐axis shows normalised power (%). Shaded areas indicate variability across samples. The AUC plots highlight spectral differences between groups across frequency bands.

Although no significant differences were observed during the light phase across all SP features, SP characteristics during the dark phase showed distinctive dissimilarities, with HA mice having a significantly higher SP duration (*p* = 0.0091, AUC = 0.283, 95% CI: 0.148–0.441, Figure 6c) and normalised amplitude (*p* = 0.0176, AUC = 0.303, 95% CI: 0.159–0.461, Figure 6d). SP amount and density showed no significant difference throughout the 24 h (Figure 6a,b). For within‐group light/dark‐phase‐wise comparisons, please refer to Figure S1.

**FIGURE 6:**
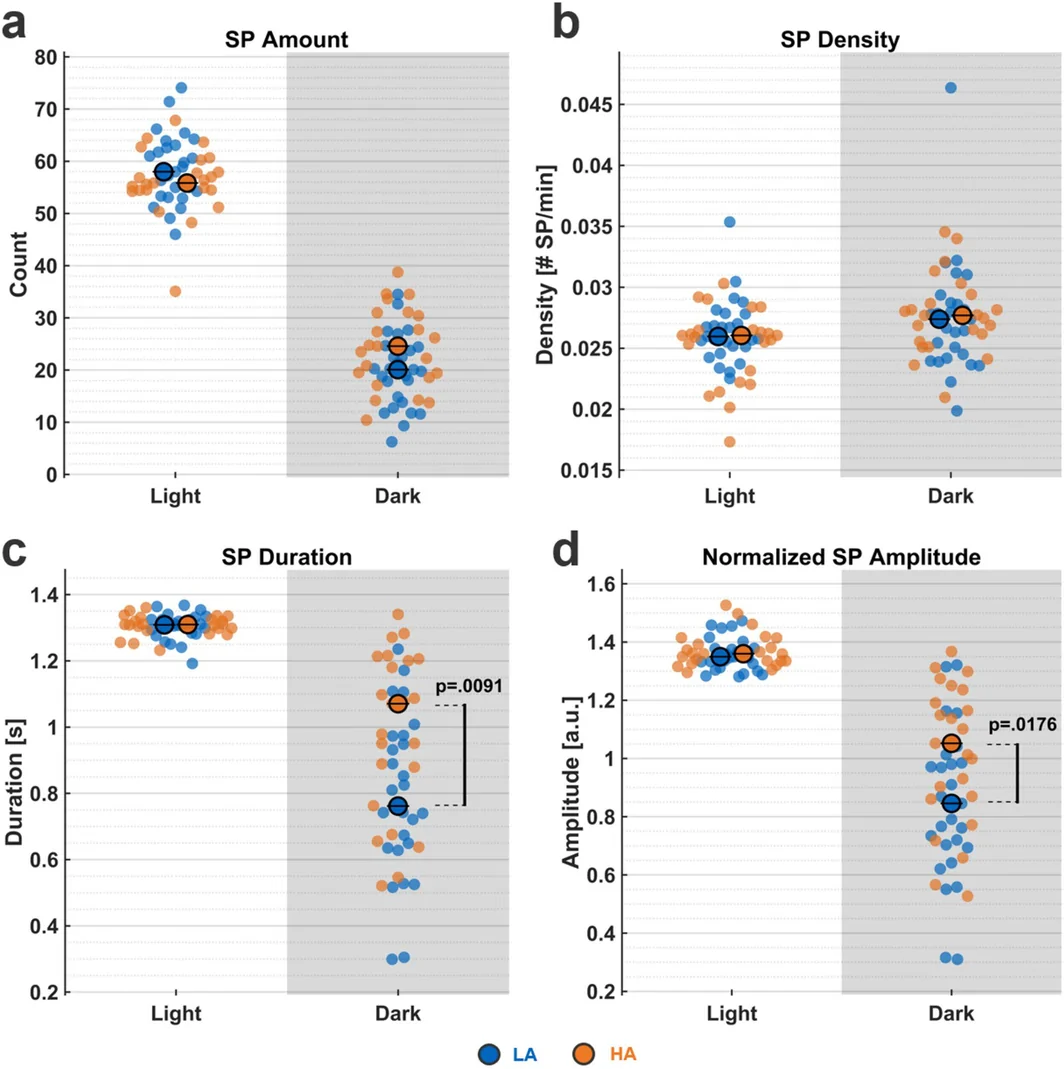
Comparison of SP characteristics between LA and HA mice across light and dark phases. (a) SP amount. The total SP count was indifferent across 24 h. (b) SP density (SP count per minute of NREMS) remained similar between LA and HA mice. (c) SP duration. HA mice had a significantly longer median SP duration in the dark phase compared to LA mice. (d) Normalised SP amplitude. In the dark phase, HA mice had a significantly higher SP amplitude relative to LA mice. Shaded areas highlight the dark phase in phase‐wise comparisons.

All remaining vigilance state proportions, bout lengths, transition percentages, latencies and spindle measures not noted above displayed no significant differences under the basal conditions tested (Table S1).

## 4 Discussion

Our study investigated whether basal sleep–wake and EEG signatures could serve as objective indicators that precede and predict the development of anxiety‐like behaviours. By classifying mice into LA and HA groups using a cued fear‐conditioning paradigm, we could show marked differences in their basal sleep architecture—particularly in REMS and NREMS—along with distinct spectral power alterations already before these animals were behaviorally tested for anxiety. Such findings suggest that EEG‐based measures, recorded under regular home‐cage conditions, may reveal inherent, trait‐like vulnerabilities to anxious states. Although further studies are required to verify the reliability and specificity of these basal EEG signatures, our data contribute to the fundamental understanding of how variations in sleep physiology might predispose or buffer against the later manifestation of anxiety‐like behaviour in mice.

One of the most prominent observations was that HA mice had reduced REMS percentages and increased NREMS to WAKE transitions during the light phase and increased NREMS during the dark phase, along with heightened power in specific high‐frequency ranges (~15–30 Hz) during REMS throughout the 24 h. These findings are consistent with previous work suggesting that trait anxiety is associated with fragmented sleep and an altered balance between NREMS and REMS (Jakubcakova et al. 2012). However, we note that our data do not imply an unprecedented mechanistic insight into anxiety itself; rather, they add evidence that basal REMS could be fundamentally intertwined with the brain's capacity to process and respond to anxiogenic stimuli.

The alterations observed in NREMS were similarly compelling. We found that HA mice displayed higher NREMS percentages during the dark period and, at the same time, exhibited reduced power in the low‐frequency *δ* range (0.5–2 Hz) in NREMS. Low frequency *δ* power is a well‐established marker of homeostatic sleep pressure and depth of NREMS (Vyazovskiy et al. 2009). Thus, lower *δ* power in HA mice may point to a deficiency in the recuperative aspects of sleep, which, in turn, might amplify their susceptibility to anxiety. Reduced delta power during NREMS may indicate a diminished homeostatic drive and a compromised capacity for restorative sleep (Goldstein and Walker 2014). Given that NREMS delta activity is associated with multiple recuperative processes essential for emotional regulation, the lower levels observed in HA mice could contribute to impaired regulation. Although our data suggest a direct link between these changes and heightened anxiety, it is worth noting that alterations in delta power can also be influenced by respiratory differences, such as variations in breathing patterns and mild respiratory events (Karalis and Sirota 2022). Consequently, insufficient deep sleep, possibly compounded by altered respiratory factors, may exacerbate anxiety vulnerability in HA mice (Goldstein and Walker 2014).

Moreover, the observed effects on SP characteristics add another layer of mechanistic depth to these findings. We observed that HA mice displayed increased SP duration and amplitude specifically during the dark phase compared to LA mice. While SPs have historically been linked to memory consolidation processes (Lüthi 2014), mounting evidence suggests they also coordinate thalamocortical communication in ways that can regulate affective states and emotional regulation (Fernandez and Lüthi 2020). If SP in the HA mice are becoming more prolonged and robust, one could speculate that this represents a maladaptive recalibration of cortical connectivity, tipping the balance towards heightened vigilance and facilitating the formation of stronger emotional (i.e., fear‐related) memories during subsequent anxiety‐inducing tasks. SP activity measurements from human patients diagnosed with PTSD showed that increased SP duration and amplitude could be associated with the over‐consolidation of negative emotional information (van der Heijden et al. 2022). Although more targeted electrophysiological and pharmacological manipulations would be necessary to fully parse this causal relationship (Natraj and Richards 2023), our results present a strong case that alterations in spindle dynamics could serve as another objective index of preexisting anxiety vulnerability. Notably, our analysis revealed that these sleep–wake and EEG alterations occurred before mice underwent FC and RET, suggesting that basal spectral markers are not simply the by‐product of these Pavlovian paradigms. Instead, these features may reflect core trait‐like differences in arousal regulation.

The current standard for quantifying rodent anxiety often hinges on measures such as freezing, open‐field avoidance or elevated plus‐maze exploration (Prut and Belzung 2003). Although these measures are highly valuable, they invariably capture anxiety only once it is expressed. As a result, they may miss early, latent signs that could guide interventions or improve experimental refinement. Our data thus bolster the rationale for incorporating basal sleep EEG recordings into future anxiety protocols, especially when investigating prophylactic treatments or mechanistic interventions that target neurophysiological processes prior to the onset of overt anxiety. From a translational standpoint, this is crucial: if basal sleep or EEG biomarkers reliably predict anxiety outcomes, a new avenue emerges for preclinical anxiety studies that avoids relying solely on ex post facto behavioural readouts.

When viewed alongside other published reports on C57BL/6 mice that link atypical sleep signatures to altered stress responses (Radwan et al. 2021), our findings converge on the concept that certain sleep characteristics, particularly in the REMS window and in the NREMS spectral domain, function as stable, trait‐like markers for vulnerability to anxiety. Though sensitivity and specificity analyses are still needed, such an objective biomarker, if consistently replicable, could markedly improve the screening of mouse populations for preclinical anxiety interventions. It also underscores the interconnectedness of sleep architecture with emotional processing circuits, highlighting how perturbations at rest can translate into exaggerated fear responses later on. These parallels resonate strongly with human literature showing that poor sleep quality often characterises individuals who later develop clinical anxiety (Cox and Olatunji 2016). Thus, despite translational challenges, our mouse data are in line with salient human findings on the role of sleep as a predictive biomarker for anxiety pathology.

Another crucial insight emerges from the NMDS of the six behavioural readout phases used for anxiety phenotyping. Rather than relying on a single phase (e.g., intertone intervals during retrieval), our approach incorporated freezing data from both FC and RET sessions and importantly, from both tone and intertone epochs. This design was motivated by the multifaceted nature of anxiety, which can manifest differently across contexts and time (LeDoux and Pine 2016) and is particularly relevant given the distinction often made between fear and anxiety (Tovote et al. 2015). Freezing elicited by the explicit tone cue is typically considered a measure of cued fear, whereas freezing during the intertone periods may reflect a more generalised, anxiety‐like state. Although related and sharing overlapping neurocircuitry, fear and anxiety are often conceptualised as involving partially distinct behavioural and neural systems. Recognising this distinction, our subsequent definition of six specific phases (tone and intertone intervals in the latter half of FC, early vs. late tone intervals in RET and early versus late intertone intervals in RET) aimed to capture fear acquisition, expression, persistence and possible extinction processes (Milad and Quirk 2012; Myers and Davis 2007).

In our NMDS results, the tone epochs from FC and RET grouped closely with one another, while the intertone intervals from both FC and RET formed a nearly orthogonal cluster. This pattern strongly suggests that the presence or absence of the auditory conditioned stimulus exerts a more dominant influence on freezing behaviour than whether the mouse is in the process of learning (FC) or recalling (RET) the aversive association. In other words, once the tone is introduced as a salient cue, its conditioned valence appears to override the difference in context between acquisition and retrieval. Conversely, intertone intervals, although not accompanied by the explicit cue, retain their own consistent ‘signature’ across FC and RET, likely reflecting contextual anxiety processes such as background threat appraisal or generalised fear responses (Phillips and LeDoux 1992).

These findings underscore the importance of evaluating multiple behavioural epochs such as tone versus intertone, early versus late and so forth, across both conditioning and retrieval sessions. A single‐phase or single‐readout approach may risk overlooking nuanced differences in how mice respond to discrete cues versus ambient contexts. Such multi‐phase, multi‐epoch analyses may align more closely with the complexity of human anxiety, which can encompass both cue‐bound (specific) and diffuse (generalised) aspects (Tovote et al. 2015). By broadening the scope of assessment, we believe to improve the ecological validity of our rodent model and enrich our understanding of how basal sleep–wake and EEG features might forecast diverse anxiety‐like patterns.

Although the present study primarily addresses the relationship between basal sleep–wake signatures and anxiety‐like behaviour, these findings may also bear relevance for clinical applications. POA has been repeatedly linked to an increased risk of postoperative complications, including pain and extended hospital stays, as well as to a heightened susceptibility to postoperative delirium (POD) (Liu et al. 2023). Converging evidence suggests that anxiety‐induced dysregulation of the hypothalamic–pituitary–adrenal (HPA) axis and altered cholinergic function may create a neurobiological substrate predisposing individuals to POD (Marcantonio 2017). In addition, perioperative sleep disturbances (Tang et al. 2023) and variations in anaesthesia type and depth (Evered et al. 2021) may compound this risk by further disrupting cognitive and stress‐response processes. Future investigations that integrate EEG‐based markers of POA with preoperative sleep parameters could thus offer novel insights, potentially paving the way for interventions designed to mitigate both anxiety and delirium outcomes.

## 5 Limitations

Several limitations of our study merit consideration. First, although our division of mice into LA and HA groups produced clear differences in basal sleep and EEG measures, factors such as circadian variability or subtle inter‐individual physiological differences may also have contributed. Second, we relied on epidural EEG rather than region‐specific recordings. Since anxiety‐related theta oscillations (4–8 Hz) typically arise from localised subcortical–cortical interactions, future work using localised electrodes or LFP recordings may yield a more detailed understanding of how basal oscillatory activity predicts anxiety‐like behaviour.

Additionally, the present study did not include a sham group receiving the conditioned stimulus without foot shock. Although this does not affect the interpretation of baseline sleep/EEG differences, as these were recorded before any experimental manipulation, a sham condition could help disentangle the contribution of the general experimental context from the conditioned fear response in future studies.

Finally, although FC is a standard method for probing anxiety‐like states, these constructs are not fully interchangeable in rodents; therefore, extrapolating our findings to broader anxiety or stress constructs in miceand especially to complex affective states in humans, should be done with caution.

## 6 Conclusion

In summary, the current study provides fundamental insights into how basal sleep and EEG differences may precede and, upon validation, predict levels of anxiety‐like behaviour in a rodent model. By extending conventional approaches to include in‐depth basal assessments of sleep architecture and EEG spectral metrics, we may move closer to an objective tool for assessing anxiety risk in rodents. Our data may also hold the potential to be transferred into a clinical predictive parameter addressing and identifying POA more systematically.

## Author Contributions

**M. V. Schmidt:** conceptualization, writing – review and editing. **A. Altunkaya:** methodology, software, data curation, investigation, writing – original draft, writing – review and editing, visualization, formal analysis, validation. **G. Rammes:** conceptualization, writing – review and editing. **T. Fenzl:** conceptualization, methodology, writing – review and editing, supervision, writing – original draft, funding acquisition, project administration. **B. Solak:** formal analysis. **K. M. Mengel:** formal analysis, data curation, investigation. **M. Kreuzer:** formal analysis, software. **G. Schneider:** resources, writing – review and editing.

## Funding

A. Altunkaya was supported by the Studienstiftung des Deutschen Volkes for AA.

## Conflicts of Interest

The authors declare no conflicts of interest.

## Supporting information


**Table S1:** Statistical analysis results for LA and HA comparison.
**Table S2:** Summary statistics, normality and bimodality tests for freezing behaviour.
**Table S3:** Comparisons of freezing proportions across six behavioural phases.
**Figure S1:** Six key behavioural measure's distribution and modality tests.
**Figure S2:** Within‐group light versus dark phase SP comparisons.

## Data Availability

The data that support the findings of this study are available from the corresponding author upon reasonable request.
