# Supplementary material for: Distinct EEG Features and Sleep/Wake Behaviour Predict Anxiety Phenotypes in Mice
Source: J Sleep Res. 2026 Jul 30;35(5):e70425. doi: 10.1111/jsr.70425 (PMC13619082; doi:10.1111/jsr.70425)
Supplement: Supplementary file 1 [file JSR-35-e70425-s001.docx]

# Supplementary Material

**Table S1:** Statistical analysis results for LA and HA comparison. For all tables showing statistics: vigilance state proportion, bout lengths, transition proportion, between-group SP comparison, within group light vs. dark phase SP comparison, and vigilance state latencies were tested for the null hypothesis that the distribution and median value of the two groups were the same. Columns 1 and 2 show the median value of group 1 followed by its sample size, whereas columns 3 and 4 stand for the median and sample size of group 2. Column 5 indicates the p-value of the corresponding statistical test. For an effect size comparison, area under the receiver-operating characteristic curve (AUROC) was employed. Column 6 shows the resulting AUC value, with lower and upper 95% confidence intervals, shown in columns 7 and 8, respectively. Mann-Whitney U test was employed for all temporal features of sleep/wake behavior other than the within-group SP comparison, where the Wilcoxon signed-rank test was used.

**Table S2:** Summary statistics, normality, and bimodality tests for freezing behavior. Descriptive statistics (median, IQR, min, max, MAD, skewness, kurtosis, CV), Shapiro-Wilk normality test p-values, and Hartigan’s Dip Test for bimodality (dip statistic, p-value) for freezing percentages across different conditioned stimulus (CS) and intertone intervals during fear conditioning (FC) and retrieval (RET) sessions.

|  | **FC Tone** $\bar{\text{CS4-5}}$ | **FC Intertone** $\bar{\text{CS4-5}}$ | **RET Tone** $\tilde{\text{CS 1-5}}$ | **RET Intertone** $\tilde{\text{CS 1-5}}$ | **RET Tone** $\tilde{\text{CS 6-10}}$ | **RET Intertone** $\tilde{\text{CS 6-10}}$ |
| --- | --- | --- | --- | --- | --- | --- |
| **Median** | 67.55 | 80.6 | 70.09 | 81.69 | 80.71 | 63.07 |
| **IQR** | 22.23 | 18.28 | 18.5 | 27.03 | 16.95 | 40.17 |
| **Min** | 22.46 | 27.87 | 44.71 | 20.18 | 44.17 | 3.66 |
| **Max** | 93.07 | 98.88 | 95.48 | 100 | 96.78 | 100 |
| **MAD** | 9.77 | 9.49 | 9.43 | 13.43 | 8.14 | 19.97 |
| **Skewness** | -0.7 | -1.02 | 0.02 | -1.08 | -0.75 | -0.28 |
| **Kurtosis** | 3.52 | 4.24 | 2.41 | 3.17 | 2.8 | 2.23 |
| **CV** | 0.14 | 0.12 | 0.13 | 0.16 | 0.1 | 0.32 |
| **p-value (Shapiro-Wilk)** | 0.048 | 0.007 | 0.906 | 0 | 0.008 | 0.116 |
| **Dip Statistic** | 0.0435 | 0.0306 | 0.0356 | 0.0494 | 0.0521 | 0.0488 |
| **p-value (Dip Test)** | 0.7081 | 0.9929 | 0.9486 | 0.4936 | 0.3986 | 0.5104 |

**Table S3:** Comparisons of freezing proportions across six behavioral phases. Results of a Kruskal-Wallis omnibus test and Dunn-Sidak-corrected post‑hoc pairwise comparisons for freezing percentages measured in: FC Tone CS4-5; FC Intertone CS4-5; RET Tone CS1-5; RET Intertone CS1-5; RET Tone CS6-10; and RET Intertone CS6-10. The omnibus test revealed a significant overall effect of condition on freezing (χ²(5) = 5.00, p < 0.0001). Post‑hoc comparisons were performed only if the omnibus test was significant; p‑values < 0.05 (in bold) indicate statistically significant differences after Dunn-Sidak correction.

| **Dunn-Sidak-corrected post-hoc comparison** | **p‑value** |
| --- | --- |
| FC Tone CS4-5 vs FC Intertone CS4-5 | 0.0343 |
| FC Tone CS4-5 vs RET Tone CS1-5 | 0.9039 |
| FC Tone CS4-5 vs RET Intertone CS1-5 | 0.0003 |
| FC Tone CS4-5 vs RET Tone CS6-10 | 0.0005 |
| FC Tone CS4-5 vs RET Intertone CS6-10 | 0.9517 |
| FC Intertone CS4-5 vs RET Tone CS1-5 | 0.8434 |
| FC Intertone CS4-5 vs RET Intertone CS1-5 | 0.9751 |
| FC Intertone CS4-5 vs RET Tone CS6-10 | 0.9904 |
| FC Intertone CS4-5 vs RET Intertone CS6-10 | 0.7553 |
| RET Tone CS1-5 vs RET Intertone CS1-5 | 0.0767 |
| RET Tone CS1-5 vs RET Tone CS6-10 | 0.1083 |
| RET Tone CS1-5 vs RET Intertone CS6-10 | 1.0000 |
| RET Intertone CS1-5 vs RET Tone CS6-10 | 1.0000 |
| RET Intertone CS1-5 vs RET Intertone CS6-10 | 0.0522 |
| RET Tone CS6-10 vs RET Intertone CS6-10 | 0.0751 |
|  |  |
| **Kruskal-Wallis omnibus (χ²(5) = 5.00)** | <0.0001 |

**Figure S1:** Six key behavioral measure’s distribution and modality tests. **(a)** Histograms of FC/RET Tone/Intertone freezing proportions. **(b)** Hartigan’s Dip Test results. Histograms show the bootstrapped distribution properties along with the empirical dip obtained from the dataset.

**Figure S2:** Within-group light vs. dark phase SP comparisons. First and second rows represent light/dark-phase-wise comparisons within the LA mice **(a-d)**, and phase-wise comparisons within the HA mice **(e-h)**, respectively. Columns show spindle amount **(a,e)**, density **(b,f)**, duration **(c,g)**, and normalized amplitude **(d,h)**. Statistical significance (p-values) is indicated where applicable. The x-axis represents experimental conditions, while the y-axis denotes the corresponding spindle parameter. Shaded areas highlight the dark phase in phase-wise comparisons.
